# Recent Advancements on Immunomodulatory Mechanisms of Resveratrol in Tumor Microenvironment

**DOI:** 10.3390/molecules26051343

**Published:** 2021-03-03

**Authors:** Gagan Chhabra, Chandra K. Singh, Deeba Amiri, Neha Akula, Nihal Ahmad

**Affiliations:** 1 Department of Dermatology, University of Wisconsin, Madison, WI 53705, USA; gchhabra@dermatology.wisc.edu (G.C.); csingh@dermatology.wisc.edu (C.K.S.); damiri@wisc.edu (D.A.); nakula@wisc.edu (N.A.); 2 William S. Middleton VA Medical Center, Madison, WI 53705, USA

**Keywords:** resveratrol, cancer, immunomodulation, tumor microenvironment, immune cells

## Abstract

Immunomodulation of the tumor microenvironment is emerging as an important area of research for the treatment of cancer patients. Several synthetic and natural agents are being investigated for their ability to enhance the immunogenic responses of immune cells present in the tumor microenvironment to impede tumor cell growth and dissemination. Among them, resveratrol, a stilbenoid found in red grapes and many other natural sources, has been studied extensively. Importantly, resveratrol has been shown to possess activity against various human diseases, including cancer. Mechanistically, resveratrol has been shown to regulate an array of signaling pathways and processes involving oxidative stress, inflammation, apoptosis, and several anticancer effects. Furthermore, recent research suggests that resveratrol can regulate various cellular signaling events including immune cell regulation, cytokines/chemokines secretion, and the expression of several other immune-related genes. In this review, we have summarized recent findings on resveratrol’s effects on immune regulatory cells and associated signaling in various cancer types. Numerous immunomodulatory effects of resveratrol suggest it may be useful in combination with other cancer therapies including immunotherapy for effective cancer management.

## 1. Introduction

Resveratrol (*trans*-3,4,5-trihydroxystilbene) is a stilbenoid that naturally occurs in many sources such as red grapes, blueberries, raspberries, rhubarb, peanuts, mulberries, and in derived products e.g., red wine [1]. It is a phytoalexin produced by various plants in response to injury or when the plant is attacked by pathogens [2]. In numerous research reports, resveratrol has been demonstrated as an anticancer, antimicrobial, and anti-inflammatory agent. Interestingly, resveratrol has been shown to possess activity against many different human diseases including obesity, diabetes, cardiovascular diseases, neurodegeneration, encephalomyelitis, multiple sclerosis, as well as different types of cancers [3,4]. Furthermore, being a natural agent, resveratrol has very low toxicity, making it a great choice for various experimental studies [5]. Resveratrol has been demonstrated to affect all the stages of cancer development (tumor initiation and promotion) and progression (angiogenesis and metastasis) [1,5,6]. Based on the available literature, we recently propagated the idea of resveratrol-based combinatorial strategies for the management of cancer [7,8]. Furthermore, resveratrol can regulate an array of signaling pathways and processes involving the reduction of oxidative stress and inflammation, inducing apoptosis, and other anticancer effects. Resveratrol also affects the respiratory chain, oncoproteins, and the expression of various regulatory genes; this includes mitochondrial functions that are connected to the tumor suppressor protein P53 [9]. In some cancers, resveratrol can act as a chemosensitizer that lowers the threshold of cell death induction. Resveratrol is also able to counteract tumor cell chemoresistance [10]. Several experimental in vitro and in vivo studies as well as clinical trials have been performed to provide evidence of preventive and therapeutic activities of resveratrol against different cancer types, which are covered by various review articles [5,11], including those published from our laboratory [4,7,8,12,13,14,15,16,17].

With regard to the immune system, resveratrol has been shown to activate immune cells such as macrophages and T-cells [18]. Specifically, resveratrol has been shown to inhibit the production of inflammatory factors via the activation of an NAD^+^-dependent deacetylase Sirtuin-1 (SIRT1) [19], which is known to be involved in various cellular processes including immune functions [20,21,22,23]. Since resveratrol can suppress inflammation through SIRT1 activation, experimental autoimmune disease models such as rheumatoid arthritis and type I diabetes have shown resveratrol’s ability to alleviate inflammatory symptoms [24]. 

Furthermore, recent advancements in cancer immunotherapy have shifted research focus from targeting the tumor itself to enhancing cancer immunity produced by the host’s immune system, leading to increased tumor-infiltrating immune cells, which can recognize and eventually destroy tumor cells. Interestingly, apart from inhibiting tumor cell proliferation and inducing apoptosis, resveratrol has also been shown to interfere with various cellular signaling events responsible for immune cell regulation, secretion of cytokines/chemokines, and regulating several other immune-related genes in the tumor microenvironment [18]. In this mini-review, first, we summarize specific anti-cancer mechanisms of resveratrol followed by a summary of recent findings on resveratrol’s immunomodulatory effects and mechanisms against cancer. We have reviewed the recent relevant literature of considerable significance suggesting resveratrol’s effects on various immune cells in tumor microenvironments including cytotoxic T-cells and dendritic cells, regulatory T cells, and tumor-associated macrophages and natural killer cells.

## 2. Resveratrol and Anticancer Effects

In 1997, Jang et al. experimented with a mouse-skin cancer model to determine the effects of resveratrol. Their results showed that resveratrol inhibited skin carcinogenesis [25]. Subsequently, various studies have demonstrated that resveratrol exerts in vitro cytotoxic and in vivo anti-tumor effects against several cancer types including breast, ovary, stomach, liver, thyroid, and prostate cancer. Aziz et al. reviewed the cancer chemopreventive properties of resveratrol in an organ-specific manner including the mechanisms involved therein [12]. It is important to note that resveratrol can impact the signal-transduction pathways that are involved in angiogenesis, metastasis, apoptosis, inflammation, and cell growth [5]. Since resveratrol modulates these critical cellular pathways, it is able to affect different stages of cancer development and progression. 

Mechanistically, resveratrol has been shown to change the proteins of the BCL2 (B-cell lymphoma 2) family and activate pro-apoptotic proteins including BAK (BCL2 antagonist/killer) and BAX (BCL2 associated X), thus leading to cell death in cancer cells [26]. Resveratrol has also been shown to induce apoptosis through inhibition of the PI3K/AKT/mTOR pathway, as well as modulation of the mitogen-activated protein kinase pathway (MAPK) in various cancer cells. It has been reported that the anti-tumor effects of resveratrol require MAPK-induced P53 activation and the induction of apoptosis. Furthermore, resveratrol has been shown to induce apoptosis in breast, prostate, ovarian, uterine, and myeloma cells [5]. Importantly, resveratrol has been shown to inhibit the proliferation of both estrogen-receptor-positive (ER-positive) and ER-negative breast cancer cells [27]. A study done by Chen et al. found that resveratrol was able to inhibit the phosphorylation of PI3K/AKT in prostate cancer cells. When resveratrol inhibits the phosphorylation of PI3K/AKT in prostate cancer cells, it results in decreased forkhead box protein (FOXO) activation [28]. Another study from our laboratory has shown that resveratrol inhibited the activation of PI3K/AKT which, in turn, resulted in modulations in BCL2 family proteins, leading to the apoptosis of androgen-responsive human prostate carcinoma cells [29]. Interestingly, it has been shown that resveratrol triggers apoptosis in human T-cell acute lymphoblastic leukemia cells mediated by inhibiting the Notch pathway as well as influencing the p53 and PI3K/AKT pathways [30].

Various studies have demonstrated that resveratrol can be a tumor-initiation suppressor by blocking the transcriptional activation of phase I and phase II cytochrome P450 enzymes (CYPs) [9]. In a clinical study, forty-two healthy volunteers were given 1 g of resveratrol for 4 weeks after baseline evaluation to determine the effects on CYPs. The results of this study suggest that resveratrol intervention was able to inhibit the phenotypic indices of cytochrome P450 enzymes CYP3A4, CYP2D6, and CYP2C9. Resveratrol clinically can interact with enzyme systems that inhibit carcinogenesis. This study concluded that resveratrol can modulate enzyme systems that are involved in carcinogenesis; however, pharmacologic doses of resveratrol could potentially lead to increased adverse drug reactions or altered drug efficacy due to the inhibition or induction of certain CYPs. Thus, lower doses should be considered for the clinical development of resveratrol for cancer prevention to minimize adverse metabolic drug interactions [31]. The inhibition of these phase I enzymes is vital because it prevents the development of any carcinogens. Resveratrol can also increase the expression and activity of various phase II enzymes, such as glutathione peroxidase and heme oxygenase. Studies have shown that resveratrol decreases the mitochondrial membrane potential [32]. Resveratrol also increases reactive oxygen species (ROS) generation, and this aids apoptosis. An increase in ROS production helps to activate the oncogenic signaling of cancer cells [33]. The overproduction of ROS and activation of these signaling pathways are important tumor-suppressive mechanisms by resveratrol that could be vital in creating anticancer strategies.

Another study has further shown that resveratrol can increase the expression and activity of quinone oxidoreductase-1 (NQO1) in human leukemia K562 cells [34]. In mouse liver-cancer Hepa 1c1c7 cells, resveratrol has been able to induce the activity of quinone reductase (QR) [35]. Resveratrol has also been shown to induce QR expression via the estrogen receptor β in breast cancer cells. Inducing QR expression via the estrogen receptor β is vital because this protects against oxidative damage to DNA. Through the activation of nuclear factor erythroid 2-related factor 2 (NRF2), resveratrol can increase the activity and expression of antioxidant and phase-II detoxifying enzymes. Further, resveratrol has been shown to upregulate the expression of heme oxygenase-1 (HO-1), which leads to the activation of NRF2 [5].

Overall, a plethora of studies, including those discussed above showing anticancer effects of resveratrol, suggest pleiotropic mechanisms of resveratrol against various cancer types. Some of these effects could be further associated with the immune-modulatory activities of resveratrol.

## 3. Recent Advancements in Resveratrol’s Immunomodulatory Activities

**Figure 1:**
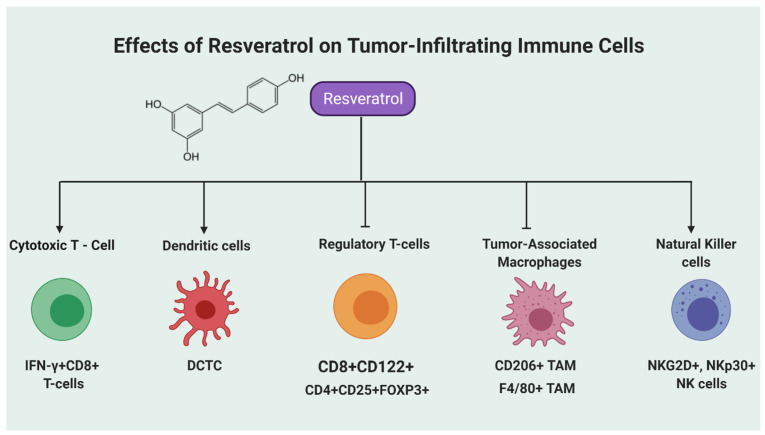
Schematic representation of resveratrol’s effects on tumor-infiltrating immune cells. Studies have shown that resveratrol either activates or suppresses immune cells in the tumor microenvironment to exert its anticancer effects. This cartoon summarizes the effects of resveratrol on specific immune cells. CTLs: cytotoxic T-cells; DCs: dendritic cells [37,38,39,40,41,42,43,44,45,46]; Tregs: regulatory T-cells; TAMs: tumor-associated macrophages [47,48,49,50,51,52,53,54,55,56,57,58,59,60,61]; NK: natural killer cells [62,63,64,65,66]. Visualization created with BioRender.com.

**Figure 2:**
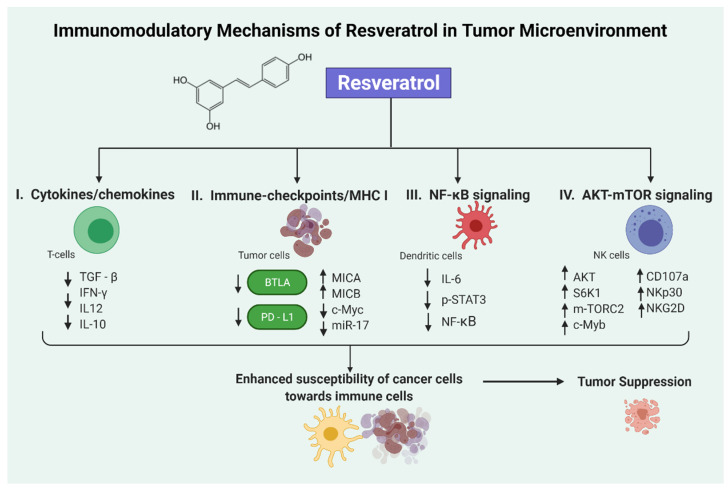
Schematic representation of resveratrol’s immunomodulatory mechanisms in tumor microenvironment. Resveratrol can regulate various cellular signaling events including immune cell regulation by modulating cytokines/chemokines secretion [45,46,53,54,58,59,65], immune-checkpoints/MHCI (major histocompatibility complex 1) [41,42,43,44], NF-κB (Nuclear factor-κB) signaling [42,46], AKT-mTOR (mammalian target of rapamycin) signaling [64] and natural killer (NK) activating receptors [65], which ultimately lead to tumor inhibition. This picture depicts the immunomodulatory mechanisms associated with resveratrol reported against cancer. Visualization created with BioRender.com.

As discussed in the introduction section, the immunomodulation of the tumor microenvironment is emerging as an important area of research for the treatment of cancer patients. The tumor microenvironment comprises various non-tumoral cells, which are majorly endothelial cells, cancer-associated fibroblasts, and immune cells. These immune cells include tumor-associated macrophages, cytotoxic T cells, natural killer cells, B cells, CD4^+^CD25^+^FOXP3^+^ regulatory T cells, dendritic cells, and myeloid-derived suppressor cells [36]. These immune cells play important roles in the pathogenesis of tumor growth and development. Interestingly, among various other natural agents, resveratrol has been used in various studies for its capability of activating the immune system to inhibit tumor cell growth and dissemination. Recently, resveratrol has been proposed as an immunomodulatory agent that can activate immune cells in the tumor microenvironment or by sensitizing cancer cells toward the cytotoxic signaling of immune cells [18]. In this section, we have focused on recently published literature showing the effects of resveratrol on different immune cells (Figure 1) as well as diverse immunomodulatory mechanisms (Figure 2) in various tumor models. 

### 3.1. Effects of Resveratrol on Cytotoxic T-Cells (CTLs) and Dendritic Cells (DCs)

Cytotoxic T-cells (CTLs) are the key immune cells that possess the capacity to kill tumor cells. The CTLs must be primed by their interactions with dendritic cells (DCs), CD4+ T-cells, and natural killer cells and further activated to form effector CTLs for killing tumor cells [37]. While DCs are known as functional antigen-presenting cells, they are capable of inducing CTLs mediated anti-tumor immune responses [38]. However, once the tumor is established, these effector CTLs are suppressed by immunosuppressive mechanisms via crosstalk between tumor cells with tumor-associated stromal cells within the tumor microenvironment, leading to tumor progression and invasiveness [39]. Furthermore, immunosuppressive signaling is associated with the upregulation of immune checkpoint mediators such as programmed death-1 receptor (PD-1), its ligand (PD-L1), and CTL-associated antigen 4 (CTLA-4). PD-1–PD-L1 signaling causes exhaustion of the effector CTLs within the tumor microenvironment, while CTLA-4 impedes priming of naive T-cells [40]. Thus, therapeutic approaches that effectively stimulate CTLs and DCs could be beneficial to treat cancer patients. In this regard, resveratrol has been used to determine its effects on CTLs and DCs in tumor microenvironment (Figure 1) utilizing different cancer models. Below, we discuss some of the recent studies in this area.

Verdura et al. recently published that resveratrol targets PD-L1 to enhance anti-tumor T-cell immunity [41]. In this study, they used the JIMT-1 cell line, which is a unique model of highly aggressive basal-like/HER2-positive breast cancer and naturally overexpressing the immunosuppressive molecule PD-L1. Resveratrol significantly promoted cytotoxic T-lymphocyte immune surveillance against breast tumor cells. Mechanistically, this study demonstrated that resveratrol targeted the immune evasion of cancer cells by directly disrupting *N*-glycan branching, leading to the dimerization of PD-L1, thus promoting the de-localization of PD-L1 to the plasma membrane. This mis-localization prevents the PD-1 interaction with PD-L1 that consequently increased the T-cell mediated immune responses against cancer cells, leading to cancer cell death. 

In another study, Lucas et al. selected breast and colorectal cancer cell lines to determine the immune-regulatory effects of resveratrol and its bio-transformed product, piceatannol, on the expression of PD-L1. This study revealed that both dietary stilbenoids (resveratrol and piceatannol), alone or in combination, increased the PD-L1 expression in Cal51 triple-negative breast cancer (TNBC) and SW620 colorectal cancer cells via histone deacetylase HDAC3/p300-mediated nuclear factor (NF)-κB signaling [42]. Another very recent study by Yang et al. determined the effects of resveratrol on PD-L1 expression and the underlying mechanism in lung cancer using human lung adenocarcinoma cell lines A549 and H1299. The results of this study suggest that resveratrol, at the dose range of pharmacologic-achievable concentrations, upregulated PD-L1 expression in lung cancer cells, and that is essential for suppression of the T-cell-mediated immune response. This study further showed that resveratrol-induced PD-L1 upregulation was mediated by activation of the WNT (Wingless-related integration site) signaling pathway via deacetylation of transcriptional factor Snail by SIRT1. In turn, Snail inhibited the transcription of destruction complex component Axin2, and it enhanced the binding of β-catenin/TCF to PD-L1 promoter to increase PD-L1 expression [43]. Overall, these studies suggested a previously unexplored immunomodulating mechanism of resveratrol, which could be explored further as a new approach to restore T-cell function by targeting the immune-checkpoint signaling PD-1/PD-L1 [41]. 

Another recent study by Lin et al. showed that resveratrol inhibited thyroid hormone-induced expression of immune-checkpoint and proliferative genes in oral cancer cells [44]. In this study, the authors have shown thyroxine induced the expression of proliferative genes as well as immune-checkpoint genes such as PD-L1 and BTLA (B- and T-lymphocyte attenuator) in human oral cancer SCC-25 and OEC-M1 cells. However, resveratrol inhibited the expression of PD-L1 and BTLA genes. Overall, this study suggests that resveratrol is able to reduce the promotive effect of thyroid hormone that activates immune-checkpoint gene expression and protects cancer to escape immune surveillance. However, it will be challenging for future research to manage the resveratrol dose to overcome the stimulatory effect of thyroid hormone in the cancer microenvironment [44].

The anticancer activities of resveratrol have been determined in ovarian carcinoma by investigating its potential effects on immunogenic cell death (ICD) utilizing both in vivo and in vitro [45]. This study showed that resveratrol treatment increased both mature dendritic cells and cytotoxic T-cells in xenograft tumors. In addition, resveratrol treatment inhibited TGF-β (transforming growth factor-β1) production and stimulated IL12p7 (interleukin 12 P7) and IFN-γ (interferon gamma) secretion. Most importantly, this study suggested that the combination of resveratrol with PD-1 antibody significantly inhibited tumor growth, while depleting CD8^+^ T cells by neutralizing antibodies markedly restored tumor progression [45].

Furthermore, Fei et al. investigated the effects of resveratrol on malignantly transformed dendritic cell line (ihDCTC) induced by glioma stem cells (GSCs) in the tumor microenvironment [46]. This study first showed that the co-culture with GSC can promote the malignant transformation of bone marrow-derived dendritic cells, and these transformed cells are more sensitive to resveratrol than the traditional chemotherapeutic drugs such as cisplatin. More importantly, resveratrol treatment significantly reduced the expression levels of IL-6, p-STAT3, and NF-κB proteins in the xenograft tissue. Overall, this study suggested that resveratrol modulated IL-6/p-STAT3/NF-κB signaling in malignantly transformed dendritic cells [46].

Taken together, the studies discussed above suggest that resveratrol modulates immune checkpoints effectively and enhances CTL- and DC-mediated immune responses against cancer cells; thus, it could be investigated in combination with clinically available immune-checkpoint inhibitors for effective cancer management.

### 3.2. Effects of Resveratrol on Regulatory T-cells (Tregs) and Tumor-Associated Macrophages (TAMs)

Regulatory T-cells (Tregs) are reported to contribute to the immunosuppressive tumor microenvironment. Importantly, Tregs are shown to suppress conventional T-cell responses and anti-tumor immunity [47]. In the context of the tumor microenvironment, CD4^+^CD25^+^FOXP3^+^ Tregs have been extensively studied [48]. The other kind of Tregs, CD8^+^CD122^+^ Tregs, have not been studied well except that the elimination of CD8^+^CD122^+^ Tregs can enhance anti-tumor immunity. Furthermore, macrophages are involved in the recognition and destruction of detrimental organisms, which can also present antigens to T-cells and instigate inflammation by releasing cytokines [49]. In the context of cancer, tumor-associated macrophages (TAMs) play a key role in cancer progression, evasion from host’s immunity, and the dissemination of cancer cells to secondary sites [50]. Traditionally, macrophages can polarize into two functional phenotypes, classically activated M1 phenotype (M1-like) and alternatively activated M2 phenotype (M2-like). TAMs, which are abundant in the tumor microenvironment, are mostly of the M2 phenotype [51]. These M2-like macrophages suppress anti-tumor immune responses and enhance tumor angiogenesis, migration, as well as invasion [52]. Here, we discuss recent studies that have shown the effects of resveratrol on Tregs and TAMs. 

Interestingly, resveratrol has been shown to reduce CD8+CD122+ Tregs and TAMs (M2 macrophages) as well as elevate IFN-γ-expressing CD8+ T cells in hepatic carcinoma (HCC). Two different HCC mouse models were utilized in this study: subcutaneous Hepa1-6 cells inoculated a tumor mouse model and an orthotopic HCC mouse model in which tumor grown from subcutaneous H22 cells were excised and implanted again into the left lobe of the liver in recipient BALB/c mice under anesthesia. Furthermore, resveratrol also downregulated inhibitory cytokines and increased effector cytokines in the mouse tumor. TGF-β1 and IL-10 levels were reduced, while TNF-α and IFN-γ were increased in the tumor microenvironment in response to resveratrol treatment. This study also showed that resveratrol inhibited STAT3 phosphorylation. Similar to tumor cells, the tumor-infiltrating immune cells, such as TAMs, have been reported to present the constitutive activation of STAT3; thus, inhibition of STAT3 activation by resveratrol demonstrates a proof-of-principle that resveratrol can indeed inhibit TAMs in the tumor microenvironment [53]. Another recent study by Davoodvandi et al. determined the therapeutic potential of resveratrol in a B16F10 mouse model of melanoma lung metastasis and analyzed tumor infiltration by Tregs. In this study, IP (intraperitoneal) injection of 40 mg/kg resveratrol led to downregulation of the immunosuppressive cytokine TGF-beta, and inhibition of the CD4+ CD25+ cell population in the spleens of B16F10 tumor-bearing mice [54]. 

Another interesting study where resveratrol has been shown to suppress the M2-like polarization of TAMs was conducted by Sun et al. [55] in lung cancer. This study also suggested that the anticancer effects of resveratrol were attributed to the inhibition of TAMs via reduction in STAT3 activation. Furthermore, utilizing a mouse lung cancer xenograft model, this study showed that resveratrol significantly inhibited lung tumor growth, which was associated with the inhibition of cell proliferation and decreased expression of p-STAT3 in tumor tissues. Importantly, resveratrol inhibited F4/80 positive cells and M2 polarization in the tumors [55]. Similarly, Kimura et al. determined the effects of resveratrol treatment on M2 macrophage activation and differentiation. They also utilized resveratrol-treated condition medium in M2 macrophages to analyze effects on vascular endothelial growth factor C (VEGF-C)-induced migration, invasion, and tube formation by human lymphatic endothelial cells (HLECs). This study suggested that resveratrol treatment inhibited the production of IL-10 and MCP1 (monocyte chemoattractant protein-1) in M2 macrophages and enhanced TGF-β1 (transforming growth factor-β1). In addition, resveratrol inhibited the phosphorylation of STAT3 in the differentiation process of M2 macrophages. Moreover, this study showed that resveratrol-treated condition medium of M2 macrophages inhibited VEGF-C-induced HLEC migration, invasion, and lymphangiogenesis [56].

Furthermore, Kim et al. recently conducted a study that showed that HS-1793, a resveratrol analogue, inhibited lymphocyte damage and immune suppression by Tregs and TAMs and enhanced the anti-tumor effects of radiation therapy in breast cancer. Various studies have shown that resveratrol derivative HS-1793 [57] has more potent anticancer activity compared to its parent compound. Few earlier in vivo studies have shown that HS-1793 exerts antitumor immune responses by controlling the balance between effector T cells and immune-suppressive cells in breast cancer [58,59]. Following this, Kim et al. had shown that HS-1793 treatment to a radiation-irradiated breast tumor mouse model significantly enhanced lymphocyte proliferation with concanavalin A stimulation and inhibited the DNA damage of lymphocytes. Treatment with this resveratrol analogue was also found to decrease the number of Tregs and reduce IL-10 and TGF-β secretion in radiation irradiated tumor-bearing mice. Furthermore, HS-1793 treatment also inhibited CD206^+^ TAM infiltration in tumor tissues in comparison to the control tumors. Overall, these findings suggest that resveratrol analogue HS-1793 can enhance anti-tumor immune responses in breast cancer [60]. 

Interestingly, resveratrol has also been investigated in combination with curcumin and demonstrated to synergistically activate host immunity and inhibit cancer development with limited side effects. This recent study suggested that a combination of mild hyperthermia treatment (mEHT) with curcumin and resveratrol significantly inhibited CT-26 tumor development growth in BALB/c mice with an increase of infiltrated F4/80+ macrophages and CD3+ T-cells. Further, in the combined treatment group, Hsp70 (heat shock protein 70) was found to be overexpressed. This study supported the idea of HSP-mediated recruiting of antigen-presenting cells (APC) since significantly higher T-lymphocyte and macrophage infiltration were found after mEHT with curcumin and resveratrol treatment [61].

Overall, these studies showed a novel immune regulatory mechanism of resveratrol reversing the immunosuppressive tumor microenvironment by diminishing Tregs and TAMs and thus, it could be further explored in vaccine-based cancer therapy.

### 3.3. Effects of Resveratrol on Natural Killer (NK) Cells

Immune escape is one of the main factors for rapid cancer progression and is related to the poor efficacy of immunotherapy [39]. The inactivation of natural killer (NK) cells contributes to the immune escape of cancer cells. These NK cells serve as an anti-tumor defense through their direct cytotoxic and indirect immune-regulatory capacities [62]. It is noteworthy that resveratrol has been shown to activate NK cells. In a 2016 review article, Leischner et al. have reviewed NK cells modulation by resveratrol for cancer prevention and treatment [63]. Below, we have discussed recently published studies analyzing the effects of resveratrol on NK cells.

Lee et al. have shown that resveratrol activated AKT by regulating mammalian target of rapamycin (mTOR) complex 2 (mTORC2) by phosphatase and tensin homolog (PTEN) and ribosomal protein S6 kinase beta-1 (S6K1). Furthermore, NK cell activation by resveratrol was more dependent on the mTOR signaling pathway than the AKT pathway. Notably, resveratrol was found to upregulate the levels of c-Myb (Myb proto-oncogene), which is a downstream transcription factor of AKT/mTORC2. Moreover, c-Myb was an essential factor for resveratrol-mediated NK cell activation when used in combination with interleukin-2 (IL-2). Overall, this study demonstrated that resveratrol was able to activate NK cells through AKT- and mTORC2-mediated c-Myb upregulation [64].

In another study, Lee et al. screened several compounds including antioxidants, vitamins, and food ingredients to identify compounds that can activate NK cells. Out of several other compounds, this study demonstrated the potential of resveratrol as an anticancer and anti-metastatic drug candidate, which may act via NK cell activation. 

Furthermore, this study demonstrated that resveratrol treatment activated NK cells most effectively and synergistically enhanced IFN-γ secretion and NK cell-mediated cytotoxicity in combination with interleukin-2 (IL-2). Importantly, resveratrol and IL-2 combination upregulated CD107a, NKp30, and NKG2D (NK receptors) expression levels on the surface of NK cells when compared with IL-2 treatment alone. Moreover, resveratrol treatment enhanced NK cell activity in human and mouse blood. Importantly, the enhanced NK cell activity by resveratrol was associated with a significant inhibition of tumor growth and metastasis in mice [65]. 

Resveratrol has also been reported to elevate the major histocompatibility complex class I chain-related proteins A and B (MICA and MICB) expression in breast cancer cells by suppressing the c-Myc/miR-17 pathway (Figure 2), thus enhancing the susceptibility of breast cancer cells toward NK cells. Interestingly, resveratrol inhibited c-Myc expression, which further downregulated the transcription of miR-17-92 cluster, thereby reducing the expression of miR-17 [66].

These studies suggest that resveratrol is a promising natural agent, which is able to effectively stimulate immune responses including via enhancing the NK cell-mediated killing of cancer cells. We have summarized the immunomodulatory effects of resveratrol in the tumor microenvironment in Table 1.

## 4. Conclusions and Future Perspectives

Based on the above-discussed studies, we conclude that the anti-tumor effects of resveratrol are not only by inhibiting cell proliferation and inducing the apoptosis of tumor cells but also in part by enhancing anti-tumor immunity and reversing the immunosuppressive tumor microenvironment. Furthermore, the effects of resveratrol on the immune system are associated with widespread health benefits for various human diseases, including cancer. Immunomodulatory effects of resveratrol have been described both in vivo and in vitro studies utilizing various types of cancer models. Based on these studies, we conclude that resveratrol favors tumor inhibitory immune cells in the tumor microenvironment, increasing the immunogenicity and thus, it could be beneficial in the combination of cancer immunotherapies. It is important to note that resveratrol modulates various cytokines and chemokines, which could be context-dependent. However, many factors need to be taken into consideration before resveratrol can be clinically in use for cancer prevention and/or therapy. Future perspectives may include the identification of new analogs and derivatives of resveratrol, which may have superior immunomodulatory properties, an ideal combination of resveratrol with other immunotherapies, and detailed evaluation of resveratrol’s immunoregulatory mechanisms and validation in clinical trials.

## Figures and Tables

**Table 1:** The immunomodulatory effects of resveratrol in the tumor microenvironment.

Cell Model	Treatment Duration	Treatment Concentration	Effects of Resveratrol	Ref
JIMT-1 breast cancer cells	5–10 h	100 μM	Significantly promoted cytotoxic T-lymphocyte immune-surveillance against breast tumor cells. Targeted the immune evasion of cancer cells by directly disrupting N-glycan branching leading to dimerization of PD-L1, which promoted de-localization of PD-L1 to the plasma membrane.	[41]
Cal51 breast cancer and SW620 colorectal cancer cells	48 h	5–100 μM	Increased PD-L1 expression via HDAC3/p300-mediated nuclear factor NF-κB signaling.	[42]
A549 and H1299 lung cancer cells	3 h	0.1–60 μM	Enhanced binding of β-catenin/TCF to PD-L1 promoter and increased PD-L1 expression. At a higher dose (>40 μM), resulted in a progressive reduction of PD-L1.	[43]
Oral cancer cells	24 h	40 μM	Inhibited thyroid hormone-induced expression of immune-checkpoint and proliferative genes. Inhibited the expression of PD-L1, BTLA genes.	[44]
Ovarian carcinoma	24–48 h	25, 50 μM	Increased both mature dendritic cells and cytotoxic T-cells in xenograft tumors. Inhibited TGF-β production and stimulated IL12p7 and IFN-γ secretion. The combination of resveratrol with PD-1 antibody significantly inhibited tumor growth, while depleting CD8+ T cells by neutralizing antibody markedly restored tumor progression.	[45]
Dendritic cells	24–72 h	10–200 μM	Reduced the expression levels of IL-6, p-STAT3 and NF-κB proteins in the xenograft tissue. Modulated IL-6/p-STAT3/NF-κB signaling in malignantly transformed dendritic cells.	[46]
Liver cancer cells	4 days, 21 days	10–40 μM	Reduced CD8+CD122+ Tregs and TAMs (M2 macrophages), and elevated IFN-γ-expressing CD8+ T cells. Downregulated inhibitory cytokines while increased effector cytokines in the mouse tumor. Inhibited STAT3 phosphorylation. TGF-β1 and IL-10 levels were reduced, while TNF-α and IFN-γ were increased.	[53]
B16F10 Melanoma cells	3 weeks	40 mg/kg	Reduced the immunosuppressive cytokine TGF-β, and inhibited the CD4+ CD25+ cell population in the spleens of B16F10 tumor-bearing mice.	[54]
Tumor-associated macrophages in lung cancer	24–48 h	20 μM	Inhibited lung tumor growth, which was associated with inhibition of cell proliferation and decreased expression of p-STAT3 in tumor tissues. Inhibited F4/80 positive cells and M2 polarization in the tumors.	[55]
Human lymphatic endothelial cells	12–24 h	5–50 μM	Resveratrol-treated condition medium of M2 macrophages inhibited VEGF-C-induced cell migration, invasion, and lymphangiogenesis.	[56]
FM3A Murine mammary carcinoma cells	24–48 h	0.31–10 μg/mL	Enhanced lymphocyte proliferation with concanavalin A stimulation and inhibited the DNA damage of lymphocytes. Decreased the number of Tregs and reduced IL-10 and TGF-β secretion in radiation irradiated tumor-bearing mice. Inhibited CD206+ TAM infiltration in tumor tissues in comparison to the control tumors.	[58]
Tumor-associated macrophages in breast cancer	24 h	1.25–5 μM	Reduced level of tumor progressive mediators by IFN-γ exposure. Exerted antitumor immune responses by controlling the balance between effector T cells and immune-suppressive cells.	[59]
CT-26 colon cancer cells	24 h	25, 50 μM	Inhibited CT-26 tumor development growth in BALB/c mice with an increase of infiltrated F4/80+ macrophages and CD3+ T-cells.	[61]
Natural killer (NK) cells	36 h	20 μM	Activated NK cells through AKT and mTORc2-mediated c-Myb upregulation.	[64]
Natural killer (NK) cells	0–48 h	20 μM	Activited NK cells, inhibited tumor growth and metastasis in mice. Enhanced NK cell activity in human and mouse whole blood. When combined with IL-2, upregulated CD107a, NKp30, and NKG2D expression levels on the surface of NK cells.	[65]
Breast cancer cells	48 h	6.25, 25 μM	Upregulated expression of MICA and MICB by suppressing c-Myc/miR-17 pathway. Increased the cytolysis by NK cells.	[66]

## Data Availability

Not applicable.
